# Deep neural networks for human microRNA precursor detection

**DOI:** 10.1186/s12859-020-3339-7

**Published:** 2020-01-13

**Authors:** Xueming Zheng, Xingli Fu, Kaicheng Wang, Meng Wang

**Affiliations:** 1 Department of Biochemistry and Molecular Biology, School of Medicine, Jiangsu University, Zhenjiang, China; 2 Jiangsu University Health Science Center, Jiangsu University, Zhenjiang, China; 3 Intensive Care Unit, HuiShan People’s Hospital of Wuxi, Wuxi, Jiangsu Province China; 4 School of Computer Science, Jiangsu University of Science and Technology, Zhenjiang, China

**Keywords:** miRNAs, DNN, Detection

## Abstract

**Background:**

MicroRNAs (miRNAs) play important roles in a variety of biological processes by regulating gene expression at the post-transcriptional level. So, the discovery of new miRNAs has become a popular task in biological research. Since the experimental identification of miRNAs is time-consuming, many computational tools have been developed to identify miRNA precursor (pre-miRNA). Most of these computation methods are based on traditional machine learning methods and their performance depends heavily on the selected features which are usually determined by domain experts. To develop easily implemented methods with better performance, we investigated different deep learning architectures for the pre-miRNAs identification.

**Results:**

In this work, we applied convolution neural networks (CNN) and recurrent neural networks (RNN) to predict human pre-miRNAs. We combined the sequences with the predicted secondary structures of pre-miRNAs as input features of our models, avoiding the feature extraction and selection process by hand. The models were easily trained on the training dataset with low generalization error, and therefore had satisfactory performance on the test dataset. The prediction results on the same benchmark dataset showed that our models outperformed or were highly comparable to other state-of-the-art methods in this area. Furthermore, our CNN model trained on human dataset had high prediction accuracy on data from other species.

**Conclusions:**

Deep neural networks (DNN) could be utilized for the human pre-miRNAs detection with high performance. Complex features of RNA sequences could be automatically extracted by CNN and RNN, which were used for the pre-miRNAs prediction. Through proper regularization, our deep learning models, although trained on comparatively small dataset, had strong generalization ability.

## Background

MiRNAs play import roles in gene expression and regulation and are considered to be important factors involved in many human diseases, e.g. cancer, vascular diseases or inflammation [1–3]. The biogenesis of miRNAs starts with the transcription of miRNA genes which forms primary miRNA hairpins (pri-miRNA). Then the pri-miRNAs were cleaved in the nucleus by RNase III enzyme Drosha, producing pre-miRNAs [4]. In an alternative pathway for miRNAs biogenesis, the pre-miRNA is from branched introns which are cleaved by debranching enzyme DBR1 [5, 6]. After transportation to cytosol by Exportin-5, pre-miRNAs are further processed into small RNAs duplexes by another RNase III enzyme Dicer [7, 8]. Finally, the duplex loads into the silencing complex, wherein most cases one strand is preferentially retained (mature miRNA), while the other strand is degraded [9].

MiRNAs can be detected using experimental methods such as quantitative real-time PCR (qPCR), microarray and deep sequencing technologies [10–12]. All the experimental methods suffer from low specificity which needs extensive normalization. Furthermore, both qPCR and microarray can only detect known miRNAs since the primers for qPCR and the short sequences on microarray need to be predesigned [13].

Due to the difficulty of discovery of new miRNAs from a genome by existing experiment techniques, many ab initio computational methods have been developed [11]. Most of these classifiers which utilize machine learning algorithms such as support vector machines (SVM), are based on the carefully selected characteristics of pre-miRNAs [14–18]. The hand-crafted features of pre-miRNAs are the most important factors for the performance of the classifiers and therefore are generally developed by domain experts [19].

CNN and RNN, the two main types of DNN architectures, have shown great success in image recognition and natural language processing [20–22]. CNN is a kind of feedforward neural networks which contains both convolution and activation computations. It is one of the representative algorithms of deep learning, which can automatically learn features from raw input features [23]. The convolution layer, consisting of a combination of linear convolution operation and nonlinear activation function, is usually followed by a pooling layer which provides a typical down-sampling operation such as max pooling [24]. Through using multiple convolution and pooling layers, CNN models can learn patterns from low to high level in the training dataset [25].

Much as CNN is born for processing a grid of values such as image, RNN is specialized for processing sequential data [22]. One of the most popular RNN layers used in practical applications is called long short-term memory (LSTM) layer [26]. In a common LSTM unit, there are three gates (an input gate, an output gate and a forget gate) controlling the flow of information along the sequence. Thus, LSTM networks can identify patterns, which may be separated by large gaps, along a sequence [27].

Lots of CNN and RNN architectures have been developed to address biological problems and shown to be successful especially in biomedical imaging processing [28–31]. Here we designed, trained and evaluated the CNN and RNN models to identify human pre-miRNAs. The results showed that our proposed models outperformed or were highly comparable with other state-of-the-art classification models and also had good generalization ability on the data from other species. Furthermore, the only information used in our models is the sequence combined with the secondary structure of pre-miRNAs. Our methods can learn automatically the patterns in the sequences avoiding the hand-crafted selection of features by domain experts, and therefore can be easily implemented and generalized to a wide range of similar problems. To the best of our knowledge, we are the first to apply CNN and RNN to identify human pre-miRNAs without the need for feature engineering.

## Results

### Model’s performance

The CNN and RNN architectures for the pre-miRNAs prediction were proposed in this study. The detailed architectures and training methods of our deep learning models were shown in the methods section. For the training/evaluation/test splitting, the models were trained on the training dataset with enough epochs, evaluated on the evaluation dataset and finally the performance on the test dataset was shown as indicated in Table 1. In the 10-fold Cross Validation (CV), the performance was tested on each of the 10-folds, while the remaining 9-folds were used for training. For conciseness, we showed that the average performance along with standard error (SE) for the 10-fold CV experiments (Table 1).

**Table 1:** Performance of the proposed models

Model (Data set partition)	Sen.(%)	Spe.(%)	F1(%)	MCC(%)	Acc.(%)
CNN (Training/Evalu./Test)	88.83	88.28	88.83	77.11	88.56
CNN (10-fold CV)	89.58 ± 4.72	84.90 ± 4.84	87.53 ± 1.38	74.72 ± 3.52	87.24 ± 1.80
RNN (Training/Evalu./Test)	85.71	91.28	88.35	77.03	88.43
RNN (10-fold CV)	85.89 ± 3.29	91.14 ± 2.75	88.09 ± 2.03	77.04 ± 3.66	88.44 ± 1.80

Note: Classification performance of different models on the testing dataset was shown as sensitivity (column 2), specificity (column 3), F1-Score (column 4), MCC (column 5) and accuracy (column 6) respectively. For the 10-fold CV, the performance was shown as mean ± standard error

As shown in Table 1, we got similar values of sensitivity (column 2), specificity (column 3), F1-score (column 4), Mathews Correlation Coefficients (MCC) (column 5) and accuracy (column 6) for these two kinds of dataset splitting strategies in each model. For both of the models, the values of sensitivity, specificity, F1-score and accuracy were mostly in the range of 80–90%, while that of MCC in 70–80%. In the CNN and RNN models, the prediction accuracy reached nearly 90%. The RNN model showed better specificity, which exceeded 90%, and poorer sensitivity (about 85%).

**Fig. 1:**
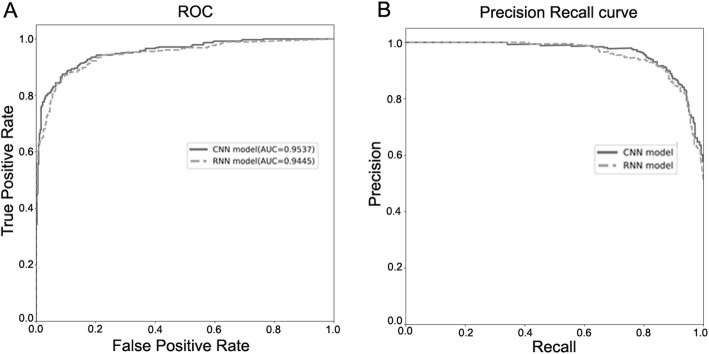
ROC and PRC of proposed DNN models. ROC (**a**) and PRC (**b**) are shown as indicated. The AUC is also shown in (**a**)

For further comparisons, we plotted the Receiver-Operating Characteristic Curves (ROC) and the precision-recall curves (PRC) of different models for the training/evaluation/test splitting. All the parameters were trained on the training dataset and all the curves were drawn based on the test dataset. As shown in Fig. 1, the CNN model performed better reaching an area under the ROC curve (AUC) of 95.37%, while the RNN model with an AUC of 94.45%. The PRC also showed similar results.

### Performance comparison with other machine leaning methods

For comparison, we referred to a newly published work done by Sacar Demirci et al. [19]. In their study, they assessed 13 ab initio pre-miRNA detection approaches thoroughly and the average classification performance for decision trees (DT), SVM and naive Bayes (NB) was reported to be 0.82, 0.82 and 0.80 respectively. Following the same dataset splitting strategy, our models were retrained on stratified and randomly sampled training dataset (70% of the merged dataset) and validated on the remaining 30% dataset. Here, we showed that the prediction results of some representative classifiers and our deep learning methods trained on the same positive and negative datasets (Table 2). As shown in the table, our models had outperformed all the best individual methods (Ding_NB,_ Ng_DT_, Bentwich_NB_, Batuwita_NB_ and Ng_NB_), and yet were not as good as most of the ensemble methods (Average_DT_, Consensus_DT_ and Consensus).

**Table 2:** Comparison of model performance on the same benchmark datasets

Model	hsa	Pseudo
This work_CNN_	96	88
This work_RNN_	90	92
Average_DT_	97	93
Consensus_NB_	86	86
Consensus_DT_	99	90
Ding_NB_	88	84
Average_NB_	83	89
Ng_DT_	89	89
Consensus	97	96
Batuwita_NB_	86	83
Bentwich_NB_	92	71
Ng_NB_	86	81

Note: Prediction results (percentage) of this work (rows 2–3) and the top ten models (rows 4–13) of the izMiR framework [19]. The values presented were true prediction rates (%) (TPR) achieved for each model and dataset. Pseudo: negative data, from the coding region of human RefSeq genes, 8492 hairpins; hsa: positive data, *Homo sapiens* miRNAs, 1881 sequences

### Classification performance on other species

Since our models were trained and tested on human dataset, we wanted to know whether the trained classifiers could be applied to other species. We fed the well-trained CNN model with the pre-miRNAs sequences from *Macaca mulatta*, *Mus musculus* and *Rattus norvegicus* to perform classification. The pre-miRNAs of these species were downloaded from miRBase (http://www.mirbase.org/) and MirGeneDB [32] (http://mirgenedb.org/). For all these three species, more than 87% pre-miRNAs from miRBase were predicted to be true, while more 99% pre-miRNAs from MirGeneDB were correctly predicted (Table 3). The relatively higher prediction accuracy of *Macaca mulatta* might result from its closer evolutionary relationship with human.

**Table 3:** Prediction accuracy on pre-RNAs datasets from other species using the CNN model trained with human data

Database	Species	# of pre-miRNAs	# of correct prediction	Accuracy (%)
miRBase release 22	*Macaca mulatta*	617	564	91.41
	*Mus musculus*	1234	1081	87.60
	*Rattus norvegicus*	495	436	88.08
MirGeneDB	*Macaca mulatta*	499	498	99.80
	*Mus musculus*	449	446	99.33
	*Rattus norvegicus*	414	411	99.28

The results showed that the proposed methods had good generalization ability on all the tested species. As we know, the quality of data is critical for deep learning. The high prediction accuracy might owe to the stricter standard for pre-miRNAs selection in MirGeneDB compared with those from miRBase.

## Discussion

In this study, we showed that both CNN and RNN could automatically learn features from RNA sequences, which could be used for computational detection of human pre-miRNAs. Because of the small size of the dataset, the data quality and the vectorization method of input sequences would have great impact on the performance of the classifier. In the initial trial of this work, we only used the sequence of RNA to perform prediction. The results showed that although our DNN models could be successfully trained on the training dataset, there were high prediction error rates in the validation dataset, indicating low generalization ability. Although we tried different model structures and regularization methods, the big generalization error could not be reduced. This problem might result from the small sample size which couldn’t be avoided. So, we combined the sequence and the secondary structure information as the input in our DNN models, which greatly minimized the generalization error. Good representations of data were essential for models’ performance, although deep learning models could learn features automatically from data.

As we know, there are lots of hyperparameters for deep learning models, which needs to be determined before training. How to tune the hyperparameters to solve specific biological problems needs to be intensely studied in the future. So, we believe that great improvement could be made to identify pre-miRNAs in the future, although the models we proposed here performed very well.

## Conclusions

In this work, we showed that both CNN and RNN can be applied to identify pre-miRNAs. Compared to other traditional machine learning methods, which heavily depend on the hand-crafted selection of features, CNN and RNN can extract features hierarchically from raw inputs automatically. In our deep learning models, we only used the sequence and the secondary structure of RNA sequences, which made it easy to implement. Furthermore, our models showed better performance than most SVM, NB and DT classifiers which were based on the hand-crafted features. To investigate the performance on other species, we tested our CNN model with pre-miRNAs sequences from other species. The results showed that our methods had good generalization ability on all the tested species especially on the datasets from MirGengDB.

## Methods

### Datasets preparation and partition

The positive human pre-miRNA dataset (Additional file 1) containing 1881 sequences was retrieved from miRBase [33, 34]. The negative pseudo hairpins dataset (Additional file 2) was from the coding region of human RefSeq genes [35], which contained 8492 sequences. The secondary structures of the RNA sequences were predicted using RNAFolds software [36] and shown in the RNAFolds column of the datasets. Both the positive and the negative datasets were widely used for training other classifiers based mostly on SVM [19]. For the balance of datasets, we randomly selected the same number of negative sequences with that of positive ones. The selected negative and positive datasets were merged together and separated randomly into training (2408 sequences), validation (602 sequences) and test (752 sequences) datasets. In the10-fold CV experiments, the merged dataset was divided into 10 segments with about the same number of sequences (376 sequences). In each experiment, nine segments were used for training while the remaining one was used for evaluating the performance of the model.

### One-hot encoding and zero padding

**Fig. 2:**
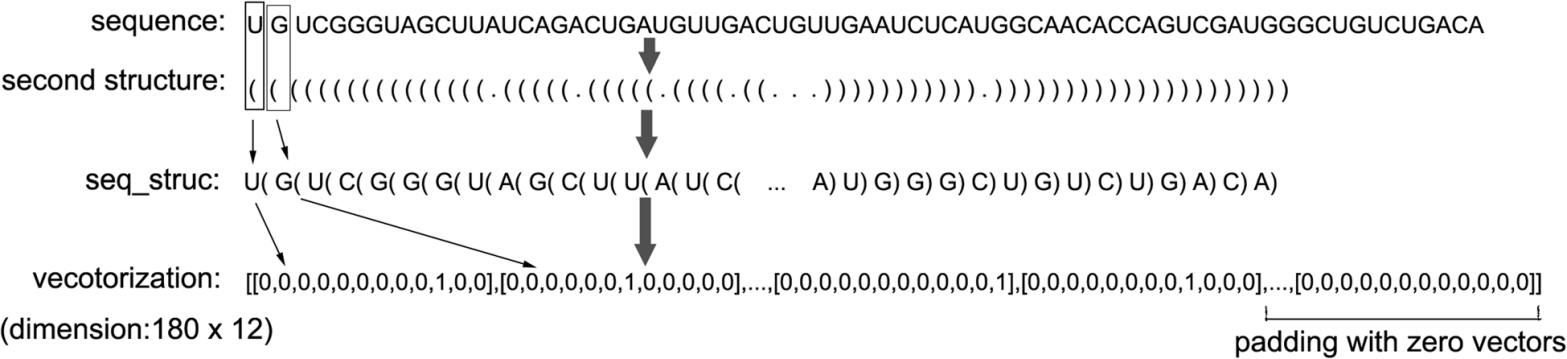
One-hot encoding and vectorization of pre-miRNA sequence. The seq_struc is the combination of nucleotide/base and the corresponding secondary structure indicated with different symbols. The left bracket “(“means paired base at 5′-end. The right bracket”)” means paired base at 3′-end. The dot “.” means unpaired base. The encoded sequence is padded with zero vectors to the length of 180

In the RNAFolds column of the supplementary datasets, the secondary structures were predicted by RNAfolds [33] and indicated by three symbols. The left bracket “(” means that the paired nucleotide/base at the 5′-end and can be paired with complimentary nucleotide/base at the 3′-end, which is indicated by a right bracket“)”, and the “.” means unpaired bases. In our deep neural networks, we only needed the sequences and the paring information. So, we merged the base (“A”, “U”, “G”, “C”) and the corresponding structure indicator (“(”, “.”, “)”) into a dimer. Since there were four bases and three secondary structure indicators, we got twelve types of dimers. The newly generated features together with the labels were stored in the new files (Additional file 3 and Additional file 4). Next, we encoded the dimers with “one-hot” encoding (twelve dimension) and padding each sequence with the zero vector to the max length of all the sequences (180). So, each sequence could be represented by a vector with the shape of 180 × 12 × 1, which was used in our supervised deep learning method (Fig. 2).

### Proposed deep neural network architecture

#### The CNN architecture for the pre-miRNAs prediction

**Fig. 3:**
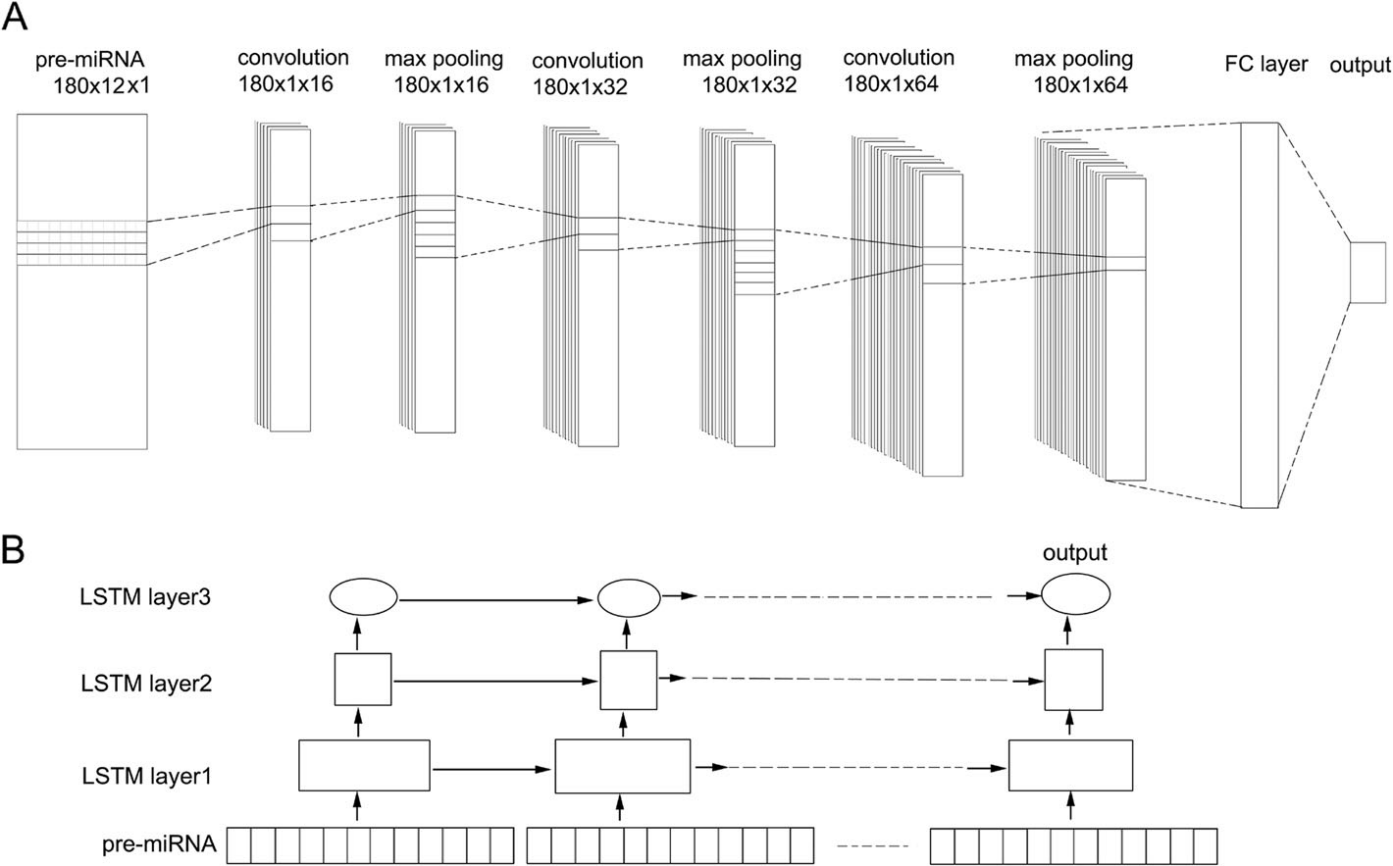
The proposed CNN and RNN architectures for pre-miRNAs prediction. **a**. CNN model. The pre-miRNA sequence is treated as a 180 × 12 × 1 vector. There are three cascades of convolution and max-pooling layers followed by two fully connected layers. The shapes of the tensors in the model are indicated by height × width × channels. FC: fully connected layer with 32 units. **b**. RNN model. Three LSTM layers with 128, 64 and 2 units respectively are shown in the RNN. The final output is passed through a softmax function with the output of probability distribution over labels. In each time step along the pre-miRNA sequence, the LSTM cells remembered or ignored old information passed along the arrows. The output was the probability distribution over the true or false labels.

The designed architecture of CNN was shown in Fig. 3a. In this model, the input sequences were first convolved by sixteen kernels with the size of four over a single spatial dimension (filters: 16, kernel size: 4), followed by the max pooling operation. Then the output tensors flowed through the second convolution layer (filters: 32, kernel size: 5) and max pooling layers, followed by the third convolution layer (filters: 64, kernel size: 6) and max pooling layers. All the max-pooling layers took the maximum value with the size of 2. After convolution and max pooling layers, all the extracted features were concatenated and passed to a fully-connected layer with 0.5 dropout (randomly ignoring 50% of inputs) for regularization in the training process. The dropout, a popular regularization method in deep learning, can improve the performance of our CNN model by reducing overfitting [37]. The last was the softmax layer whose output was the probability distribution over labels.

#### The RNN architecture for the pre-miRNAs prediction

In the recurrent neural networks (RNN) model, three LSTM layers with 128, 64 and 2 units respectively were used to remember or ignore old information passed along RNA sequences. Each LSTM unit is comprised of the following operations, where W and U are parameter matrices and b is a bias vector [27].

input gate: i_t_ = sigmoid (W_i_x_t_ + U_i_h_t-1_ + b_i_).

forget gate: f_t_ = sigmoid (W_f_x_t_ + U_f_h_t-1_ + b_f_).

transformation of input: c_in_t_ = tanh(W_c_x_t_ + U_c_h_t-1_ + b_c_).

state update: c_t_ = i_t_ · c_in_t_ + f_t_ · c_t-1_.

h_t_ = o_t_ · tanh(c_t_).

output gate: o_t_ = sigmoid (W_o_x_t_ + U_o_h_t-1_ + V_o_c_t_ + b_o_).

For avoiding overfitting, the LSTM layers were regularized with randomly ignoring 20% of the inputs. The output tensors of the last LSTM layer were then passed through the softmax layer which gave the predicted probability over each label (Fig. 3b).

### Model training

The loss function we used is the cross entropy between the predicted distribution over labels and the actual classification [38]. The formula is as follows.

1 $$ \mathrm{Cross}-\mathrm{entropy}=-\sum \limits_{\mathrm{i}=1}^{\mathrm{n}}{\mathrm{y}}_{\mathrm{i}}\log {\mathrm{s}}_{\mathrm{i}} $$

(n: the number of labels, y_i_: the actual probability for label i, s_i_: predicted probability for label i).

The aim of our machine learning was to minimize the mean loss by updating the parameters of the models. The models were fed by the training dataset and optimized by Adam algorithm [39]. The training processes were not stopped until the loss did not decrease any more. During the training process, the generalization error was also monitored using validation dataset. Finally, the learned parameters as well as the structures were stored.

### Methodology evaluation

After training, we calculated the classifier performance on the test dataset in terms of sensitivity, specificity, F1-Score, MCC and accuracy. (TP: true positive, TN: true negative, FP: false positive, FN: false negative).

Sensitivity:

2 $$ \mathrm{Sen}.=\frac{\mathrm{TP}}{\mathrm{TP}+\mathrm{FN}} $$

Specificity:

3 $$ \mathrm{Spe}.=\frac{\mathrm{TN}}{\mathrm{TN}+\mathrm{FP}} $$

F1-Score:

4 $$ \mathrm{F}1=\frac{2\ast \mathrm{TP}}{2\ast \mathrm{TP}+\mathrm{FP}+\mathrm{FN}} $$

MCC:

5 $$ \mathrm{MCC}=\frac{\mathrm{TP}\ast \mathrm{TN}-\mathrm{FP}\ast \mathrm{FN}}{\sqrt{\left(\mathrm{TP}+\mathrm{FN}\right)\ast \left(\mathrm{TN}+\mathrm{FP}\right)\ast \left(\mathrm{TN}+\mathrm{FN}\right)\ast \left(\mathrm{TP}+\mathrm{FP}\right)}} $$

Accuracy:

6 $$ \mathrm{Acc}.=\frac{\mathrm{TP}+\mathrm{TN}}{\mathrm{TP}+\mathrm{TN}+\mathrm{FP}+\mathrm{FN}} $$

Also, we plotted the ROC with the AUC and PRC for the training/evaluation/test splitting. With decreasing thresholds on the decision function used, corresponding false positive rates (FPR), TPR and precisions, recalls were computed. ROC curves were drawn based on a series of FPR and TPR, while PRC were based on precisions and recalls.

### Implementation and availability

The implemented dnnMiRPre was well trained on the models using the training dataset and can be used to predict whether the input RNA sequence is a pre-miRNA. The dnnMiRPre’s source code, which was written in Python with Keras library, is freely available through GitHub (https://github.com/zhengxueming/dnnPreMiR).

## Supplementary information

**Additional file 1.** Human pre-miRNA.

**Additional file 2.** Pseudo hairpins.

**Additional file 3.** Human pre-miRNA with generated features.

**Additional file 4.** Pseudo hairpins with generated features

## Data Availability

Models and datasets are made freely available through GitHub (https://github.com/zhengxueming/dnnPreMiR).

## Abbreviations

AUC: Area under the ROC Curve
CNN: Convolutional Neural Networks
CV: Cross Validation
DNN: Deep Neural Networks
DT: Decision Trees
FN: False Negative
FP: False Positive
FPR: False Positive Rates
LSTM: Long Short-Term Memory
MCC: Matthews Correlation Coefficient
miRNAs: MicroRNAs
NB: Naive Bayes
PRC: Precision-Recall Curves
pre-miRNA: MiRNA precursor
pri-miRNA: Primary miRNA hairpins
qPCR: Quantitative real-time PCR
RNN: Recurrent Neural Networks
ROC: Receiver-Operating Characteristic Curves
SE: Standard Error
SVM: Support Vector Machines
TN: True Negative
TP: True Positive
TPR: True Positive Rates
